# *Tet2*-mediated clonal hematopoiesis modestly improves neurological deficits and is associated with inflammation resolution in the subacute phase of experimental stroke

**DOI:** 10.3389/fncel.2024.1487867

**Published:** 2024-12-17

**Authors:** Megan A. Evans, Nicholas W. Chavkin, Soichi Sano, Hanna Sun, Taneesha Sardana, Ramya Ravi, Heather Doviak, Ying Wang, Yoshimitsu Yura, Ariel H. Polizio, Keita Horitani, Hayato Ogawa, Karen K. Hirschi, Kenneth Walsh

**Affiliations:** ^1^Hematovascular Biology Center, Robert M. Berne Cardiovascular Research Center, University of Virginia School of Medicine, Charlottesville, VA, United States; ^2^Department of Cell Biology, University of Virginia School of Medicine, Charlottesville, VA, United States

**Keywords:** *TET2*, CHIP, ischemic stroke, cerebral ischemia, inflammation, mouse

## Abstract

**Introduction:**

Recent work has revealed that clonal hematopoiesis (CH) is associated with a higher risk of numerous age-related diseases, including ischemic stroke, however little is known about whether it influences stroke outcome independent of its widespread effects on cardiovascular disease. Studies suggest that leukocytes carrying CH driver mutations have an enhanced inflammatory profile, which could conceivably exacerbate brain injury after a stroke.

**Methods:**

Using a competitive bone marrow transplant model of *Tet2*-mediated CH, we tested the hypothesis that CH would lead to a poorer outcome after ischemic stroke by augmenting brain inflammation. Stroke was induced in mice by middle cerebral artery occlusion and neurological outcome was assessed at acute (24 h) and subacute (14 d) timepoints. Brains were collected at both time points for histological, immunofluorescence and gene expression assays.

**Results:**

Unexpectedly, *Tet2*-mediated CH had no effect on acute stroke outcome but led to a reduction in neurological deficits during the subacute phase. This improved neurological outcome was associated with lower levels of brain inflammation as evidenced by lower transcript levels of various inflammatory molecules alongside reduced astrogliosis.

**Discussion:**

These findings suggest that *Tet2*-mediated CH may have beneficial effects on outcome after stroke, contrasting with the conventional understanding of CH whereby leukocytes with driver mutations promote disease by exacerbating inflammation.

## Introduction

Clonal hematopoiesis (CH) is an age-associated phenomenon whereby a substantial portion of an individuals’s blood cells are derived from a dominant hematopoietic stem cell (HSC) clone. This condition typically arises due to a somatic mutation occurring within a driver gene of a HSC, which thereby provides the mutant HSC with a competitive advantage over other HSCs. As a result, the mutation can expand and propagate through the hematopoietic system where it is carried by leukocyte progeny in the peripheral blood. Although numerous driver gene mutations have been associated with CH in the elderly, they most frequently occur in the epigenetic regulator genes, *DNMT3A*, *TET2* and *ASXL1* (Jaiswal et al., 2014; Genovese et al., 2014). Recently, CH has emerged as a novel risk factor for numerous age-related diseases, particularly cardiovascular diseases (Jaiswal et al., 2014, 2017; Yu et al., 2021; Dorsheimer et al., 2019; Pascual-Figal et al., 2021; Agrawal et al., 2022; Kim et al., 2021; Zekavat et al., 2023), and studies using murine models have suggested this relationship is causal where driver mutations can directly contribute to disease by augmenting the inflammatory profile of leukocytes (Fuster et al., 2017; Sano et al., 2018a, b; Yura et al., 2021; Fuster et al., 2020; Liu et al., 2023; Sano et al., 2019).

In 2014, a large epidemiological study made the unexpected discovery that individuals with CH have a 2.6-fold increase in the risk of ischemic stroke (Jaiswal et al., 2014). Since this discovery, follow up studies have been performed and have begun to shed further light on this association. Specifically, it has been reported that individuals with mutations in the CH driver gene, *TET2*, display an increased risk of ischemic stroke (Bhattacharya et al., 2021). Moreover, it has been documented that CH is associated with large artery stroke alongside recurrent vascular events and all-cause mortality in first-ever ischemic stroke patients (Arends et al., 2023). However, these studies assessed ischemic stroke risk and major adverse events, which are largely driven by underlying cardiovascular disease, particularly atherosclerosis. Atherosclerosis, along with several others stroke risk factors, has been associated with CH and experimental studies, including those from our group, suggest that the pathological features these conditions can be worsened by CH driver mutations (Jaiswal et al., 2017; Zekavat et al., 2023; Fuster et al., 2017, 2020; Liu et al., 2023, 2024; Edelmann et al., 2018; Rauch et al., 2023; Polizio et al., 2024). In line with this, a recent study observed that both the prevalence and complexity of atherosclerotic plaques was higher in young ischemic stroke patients with CH compared to those without (Mayerhofer et al., 2023). Thus, the observed increase in stroke incidence and recurrent vascular events in individuals with CH is likely indicative of underlying risk factors, such as atherosclerosis, possibly promoted by CH per se. Less is known about the direct effects that CH has on neurological outcome after ischemic stroke, which, to a greater degree, is governed by pathophysiological mechanisms occurring within the brain after an ischemic insult. Given that individuals with CH have an increased risk of ischemic stroke (Jaiswal et al., 2014; Bhattacharya et al., 2021), an important next step would be to establish if CH directly contributes to neurological outcomes after stroke.

In this study we assessed the impact of CH on outcomes after experimental ischemic stroke. We used a stroke model devoid of cardiovascular risk factors and other comorbidities to more fully understand if CH has direct effects on brain injury after ischemia without the potential confounding effects of systemic disease. Since it has been reported that individuals with hematopoietic *TET2* loss-of-function mutations have a higher risk of ischemic stroke (Bhattacharya et al., 2021), we used *Tet2* as a test driver gene for our study. Through analysis of ischemic stroke outcome at two distinct timepoints, we observed that *Tet2*-mediated CH had no effect on acute outcome measures but surprisingly led to a modestly improved neurological outcome at 14 d post-stroke. This improved outcome was associated with lower levels of brain inflammation. These findings suggest that *Tet2* mutant leukocytes may have protective actions in the brain after ischemia, contrasting with the traditional view of CH that has been shaped by studies of systemic disease.

## Materials and methods

### Animals

All animal procedures were approved by the University of Virginia Animal Use and Care Committee (protocol number 4205). C57Bl6 background *Tet2*-deficient (JAX stock: 023359) and C57Bl6 congenic Pep Boy (B6.SJL-Ptprca Pepcb/BoyJ; JAX stock: 002014) mice were obtained from Jackson Laboratories. Breeding colonies of these strains were maintained at the University of Virginia animal vivarium and genotyping was performed according to Jackson Laboratories standard protocols. Mice were housed in specific pathogen–free cages, under a 14 h light/10 h dark cycle and had free access to water and food pellets. Male mice were used for *in vivo* experiments to minimize potential confounding effects of the estrous cycle on stroke outcome.

### Bone marrow transplantation

For stroke studies, we sought to use a model of CH which avoids radiation to the brain, which could conceivably lead to damage of the local brain-immune niche. To do this, we used a competitive bone marrow transplantation (BMT) approach with head-shielded radiation which has been previously described in detail (Park et al., 2021). Eight- to twelve-week-old irradiated CD45.1 Pep Boy recipient mice were transplanted with suspensions of unfractioned bone marrow (BM) cells containing either 10% CD45.2 *Tet2*^−/−^ cells and 90% CD45.1 *Tet2*^+/+^ cells (denoted as *Tet2*-KO) or 10% CD45.2 *Tet2*^+/+^ cells and 90% CD45.1 *Tet2*^+/+^ cells (denoted as WT). BM cells were isolated from femurs, tibias, pelvises and humeri from 8 to 14 week donor mice following euthanasia. To limit mobility during radiation, mice were anesthetized with a mixture of ketamine (100 mg/kg) and xylazine (10 mg/kg) in sterile saline and placed in a 50 mL conical tube restrainer. The restraining tube was then placed inside a custom-made lead shield (Nuclead Co. Inc., MA, USA) measuring 1.5 in. thick. Facing perpendicular to the radiation source, mice were exposed to two doses of 5.5 Gy radiation (11 Gy in total) ~18 h apart using a ^137^Cesium-source irradiator (Shepherd Associates). After the second radiation dose, each recipient mouse was retro-orbitally injected with 4–5×10^6^ BM cells, as detailed above. Sterilized caging, diet gel and antibiotic supplemented water (5 mM sulfamethoxazole, 0.86 mM trimethoprim) were provided for the first 14 d post-irradiation to prevent dehydration and infection that may occur during this post-irradiation period. Cages of mice (i.e., those containing littermates), usually and wherever possible, housed a mixture of mice transplanted with WT and *Tet2*-KO cells to ensure genetic differences of recipient mice were minimized between groups.

### Mouse model of stroke

Focal cerebral ischemia was induced by transient middle cerebral artery occlusion (MCAO), as previously described with some modifications (Evans et al., 2018a, b). Ischemia was produced in anesthetized mice (ketamine: 80 mg/kg plus xylazine: 10 mg/kg in sterile saline i.p.) by occlusion of the right middle cerebral artery (MCA) using a 6.0 silicone-coated monofilament (Doccol Corporation, Redlands, CA, USA). All filaments had a 1–2 mm silicone coating length and were either 0.23 mm in diameter for animals ≥22 g and 0.21 mm in diameter for animals <22 g, as dictated by preliminary studies. All filaments were marked with a gold permanent marker (8 mm from the silicone tip), which was used as a guide for insertion into the internal carotid artery (ICA). Successful occlusion and reperfusion were confirmed by transcranial laser-Doppler flowmetry (Moor Instruments, USA) with the probe placed 2 mm posterior and 5 mm lateral to Bregma. Rectal temperature was monitored and maintained at 37.0 ± 0.5°C throughout the entire procedure with the use of a heated mat (Stoelting Co, Wood Dale, IL, USA). MCAO was sustained for 30 min and then the filament was retracted and removed to allow for complete reperfusion. Immediately following reperfusion, mice received 1 mL of sterile saline (s.c.) and buprenorphine (3.25 mg/kg s.c.; Ethiqa XR, Fidelis, NJ, USA). Neck and headwounds were closed with sutures and/or superglue and animals were allowed to recover in heated cages. Once mobile, mice were returned to clean home cages, which were placed partially on a heat mat. Cages were kept partially on the heat mat for the first 3 days post-stroke because the likelihood of hypothermia is greatest when mobility is limited. Mice were provided wet mash and/or hydrogel, as required throughout the experiment. Mice were only excluded from the study if: [1] >0.2 mL of blood was lost; [2] a subarachnoid hemorrhage occurred; [3] they died during the surgical procedure to induce cerebral ischemia; [4] cerebral blood flow failed to drop following insertion of the monofilament; [5] if no reperfusion occurred following removal of the filament; [6] if infarct volumes were <10 mm^3^ (WT: *n* = 1, *Tet2*-KO: *n* = 0). Sham surgeries were also performed whereby the carotid arteries were exposed but left undisturbed.

### Hematopoietic cell parameter and flow cytometric analysis of peripheral blood

Peripheral blood was collected from all mice to evaluate levels of donor cell chimerism and ensure stable hematological parameters prior to experiments. For mice undergoing surgery, blood samples were collected a minimum of 4 d prior to ensure that it did not affect surgical outcomes. Samples were obtained from the retroorbital plexus using heparin-coated capillary tubes (Fisher Scientific) and collected in K2EDTA-added microtainer blood collection tubes (BD Biosciences). Hematopoietic parameters were analyzed using the Element HT5 Veterinary Hematology Analyzer (Heska, CO, USA). To confirm successful transplantation of donor cells and determine levels of mutant clone expansion, flow cytometry was performed. Samples were prepared as previously described (Yura et al., 2021) using antibodies listed in Supplementary Table 1. Samples were acquired on BD LSRFortessa Cell Analyzer (BD Biosciences) and data were analyzed using FlowJo software (Tree Star Inc., OR, USA). The gating strategy used for analysis was similar to that reported previously (Wang et al., 2020).

### Analysis of post-stroke functional outcome

Post-stroke functional outcomes were analyzed by using a neurological deficit score as previously described with some modifications (Evans et al., 2018b). In brief, a deficit score was assigned to an animal whereby 0 = normal motor function, 1 = flexion of torso and contralateral forelimb when lifted by the tail, 2 = circling to the contralateral side when held by the tail on a flat surface but normal posture at rest, 3 = leaning to the contralateral side at rest, 4 = no spontaneous motor activity/barrel rolling, 5 = death. Due to modifications we made to the stroke model to improve survival, as detailed above, we observed that a portion of mice were still presenting with minimal neurological deficits at 72 h post-stroke. Therefore, to assess the impact of *Tet2*-mediated CH on stroke recovery at 14 d, we only included animals presenting with a substantial neurological deficit (at least 3) at 72 h post-stroke. It should be noted that prior to omission of these mice, there were no significant differences in median score between the two groups (WT = 2 vs. KO = 3, determined by Mann–Whitney test). Further, while we acknowledge, that it has been documented that this scale is only accurate acutely after stroke (Ruan and Yao, 2020; Li et al., 2004), our preliminary observations noted that a portion of mice of the Pep Boy strain display gross neurological deficits, such as leaning and/or circling at 14 d post-MCAO and beyond. Therefore, we chose to use this test at the subacute timepoint of our analysis.

### Infarct and lesion analysis

Mice were euthanized by inhalation of isoflurane and the brain was immediately removed, frozen in liquid nitrogen, and stored at −80°C. Evenly spread (separated by ~420 μm) coronal sections (30 μm thick) were obtained spanning the infarct, and thaw-mounted onto poly-L-lysine coated glass slides, were stained with thionin (0.1%) to delineate the infarct, and infarct volume was calculated as described previously (Evans et al., 2018b). For lesion analysis at 14 d post-stroke, where swelling had subsided and many brains showed signs of atrophy, we did not correct for edema and instead we used the formula: CIV = RIA × (thickness of section + distance between sections).

### Quantitative RT-PCR

RNA was extracted from brains harvested at 24 h or 14 d following stroke or sham surgery as previously described (Evans et al., 2018b). Subsequently, cDNA conversion was performed using the High-Capacity cDNA Reverse Transcription Kit (Applied Biosystems). Quantitative RT-PCR was performed with *Power* SYBR Green Master Mix (Applied Biosystems) in a QuantStudio 6 Flex PCR system. Primers for the reaction are listed in the Supplementary Table 2. Data were normalized to the housekeeping gene (β-actin) and calculated as change in fold expression relative to the sham control or contralateral hemisphere using the formula: fold-change = 2^−ΔΔCt^.

### Bulk RNA-sequencing

RNA was extracted from brains using the methods described above and total RNA was sent to Azenta Life Sciences (formally Genewiz) for sequencing. Detailed methods of sequencing and data analysis can be found in Supplementary material.

### Immunofluorescent staining

Immunofluorescent staining was performed on 10 μm fresh frozen sections collected from six brain regions (Bregma +0.06, −0.78, −1.2, −1.62, −2.04, −2.46 mm) at 24 h and 14 d post-stroke. Detailed methods of staining, imaging and analysis are provided in Supplementary material.

### Statistical analyses

Data are presented as mean ± standard error of the mean (SEM), with the exception of neurological deficit scores, which are presented as median. Statistical analyses were performed using GraphPad Prism version 10.0 (GraphPad Software Inc. San Diego, CA, USA). For data with one experimental variable, between-group comparisons were compared using a Student’s unpaired *t* test (two-tailed) for two groups. For data with two independent variables, groups were compared using a two-way ANOVA followed by a Sidak post-hoc test. Neurological deficit scores were compared using a Mann–Whitney U test for two groups or a Kruskal–Wallis test with Dunn’s multiple comparison tests for more than two groups. Cerebral blood flow data were compared using multiple *t* tests for ischemia and reperfusion phases. Statistical significance was accepted if *p*-values or adjusted *p*-values, where appropriate, were <0.05.

## Results

### *Tet2*-mediated clonal hematopoiesis model characteristics

**Figure 1:**
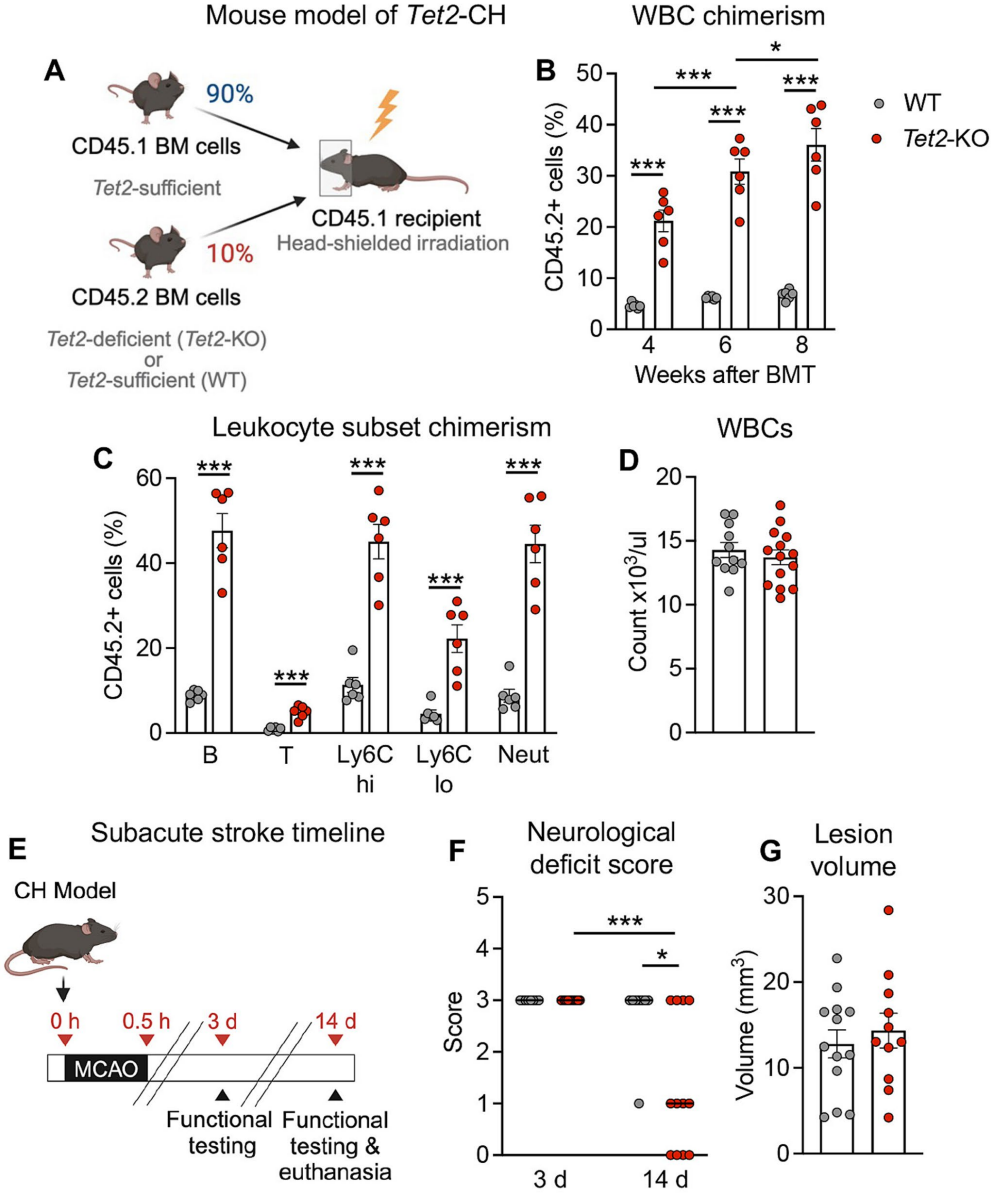
*Tet2*-mediated clonal hematopoiesis leads to fewer neurological deficits at 14 d post-stroke. **(A)** Schematic depicting the creation of the *Tet2*-clonal hematopoiesis (CH) model used for stroke studies. Following head-shielded irradiation, CD45.1 expressing recipient mice were transplanted with a mixture of donor bone marrow containing 90% of cells from CD45.1 *Tet2*-sufficient donors and 10% of cells from CD45.2 *Tet2*-KO (or wildtype; WT) donors. **(B)** Percentage of CD45.2 donor white blood cells (WBC) in peripheral blood at 4-, 6- and 8-weeks post-bone marrow transplant (BMT) with either WT or *Tet2*-KO cells (*n* = 6 per group) as determined by flow cytometry. **(C)** Percentage of CD45.2 donor cells within major peripheral blood cell lineages (left to right: B cells, T cells, Ly6Chi monocytes, Ly6Clo monocytes, neutrophils) at 8 weeks post-BMT with either WT or *Tet2*-KO cells (*n* = 6 per group) as determined by flow cytometry. **(D)** Total number of peripheral WBCs at 8 weeks post-BMT with either WT or *Tet2*-KO cells (WT: *n* = 11; *Tet2*-KO: *n* = 14). **(E)** Experimental timeline for subacute stroke experiments. At 8–10 weeks after BMT, mice were subjected to middle cerebral artery occlusion (MCAO) to induce stroke, and outcomes were assessed 3 and 14 d later. **(F)** Neurological deficit scores at 3 and 14 d post-stroke from mice transplanted with WT or *Tet2*-KO bone marrow cells (WT: *n* = 8 and *Tet2*-KO: *n* = 12). **(G)** Resolved lesion volumes at 14 d post-stroke in mice transplanted with WT or *Tet2*-KO bone marrow cells (WT: *n* = 13 and *Tet2*-KO: *n* = 11). Data are presented as mean ± SEM, except for neurological deficit scores which are presented as median. Statistical comparisons were made using a two-way ANOVA followed by Sidak post-hoc test **(B)**, Student’s unpaired *t* test **(C,D,G)**, Kruskal–Wallis test with Dunn’s multiple comparisons test **(F)**. *p < 0.05, ***p < 0.001.

To model CH in mice, a competitive BMT approach was used, as before (Fuster et al., 2017; Sano et al., 2018b). During the irradiation procedure, which facilitates donor cell engraftment, we used a shielding approach to avoid injury to the brain, like that previously described (Gliem et al., 2012; Shichita et al., 2017). Irradiated CD45.1-expressing *Tet2*-sufficient recipient mice were transplanted with a mixture of BM cells comprised of 10% of cells from CD45.2-expressing *Tet2*-deficient (*Tet2*-knock out; *Tet2*-KO) donors (or wildtype [WT] littermates as controls) and 90% of cells from CD45.1-expressing *Tet2*-sufficient donors (Figure 1A). This approach resulted in the expansion of CD45.2+ *Tet2*-KO donor cells, measuring ~21% of total white blood cells at 4 w post-BMT and increasing to ~35% by 8 w post-BMT (Figure 1B). Like previous studies (Fuster et al., 2017; Sano et al., 2018b), *Tet2*-KO donor cells expanded into all major blood cell lineages and there were no differences in the numbers of white blood cells, platelets or hemoglobin between mice transplanted with WT and *Tet2*-KO BM cells (Figure 1C and Supplementary Figures 1A,B). Body weight was also not different between the two groups (Supplementary Figure 1C). Given these findings, we chose to perform stroke surgeries between 8 and 10 w post-BMT, where we reasoned that the percentage *Tet2*-KO donor cells in the blood would be sufficient to potentially reveal any biological effect in stroke, in the absence of hematological differences between the two groups.

### *Tet2*-mediated clonal hematopoiesis does not impact acute stroke outcome, but improves neurological deficits at 14 days

As the injury response to stroke is a dynamic process with distinct mechanisms contributing at acute and subacute phases (Mergenthaler et al., 2020), we examined the effect of *Tet2*-mediated CH on outcomes during both phases. At 24 h post-stroke (Supplementary Figure 2A), it was found that there were no differences in neurological outcomes between mice transplanted with WT BM and those transplanted with *Tet2*-KO, with both groups presenting with a similar neurological deficit score (Supplementary Figure 2B). Consistent with these observations, cortical, subcortical and total infarct volumes were not different between the two groups (Supplementary Figures 2D,E). There were also no differences in edema volume (Supplementary Figure 2F). Importantly, measurements of cerebral blood flow during stroke surgeries showed that the extent of ischemia and reperfusion were similar between mice that received WT BM cells and those that received *Tet2*-KO (Supplementary Figure 2B).

To examine functional recovery during the subacute phase of stroke, we examined mice that presented with a substantial neurological deficit (a score of at least 3) at 3 d post-stroke and reexamined mice again at 14 d (Figure 1E). Mice transplanted with *Tet2*-KO cells displayed a significantly lower neurological deficit score at 14 d compared to that observed at 3 d, and this was significantly lower than that observed in mice transplanted with WT cells at this time point (Figure 1F). In contrast, mice transplanted with WT cells did not display an improvement in neurological deficit score between 3 and 14 d (Figure 1F). Consistent with findings at the 24 h time point, it was found that the resolved lesion volumes were similar between mice transplanted with WT and *Tet2*-KO BM cells (Figure 1G).

### *Tet2*-mediated clonal hematopoiesis is associated with a lower level of inflammation and astrogliosis in the subacute phase post-stroke

**Figure 2:**
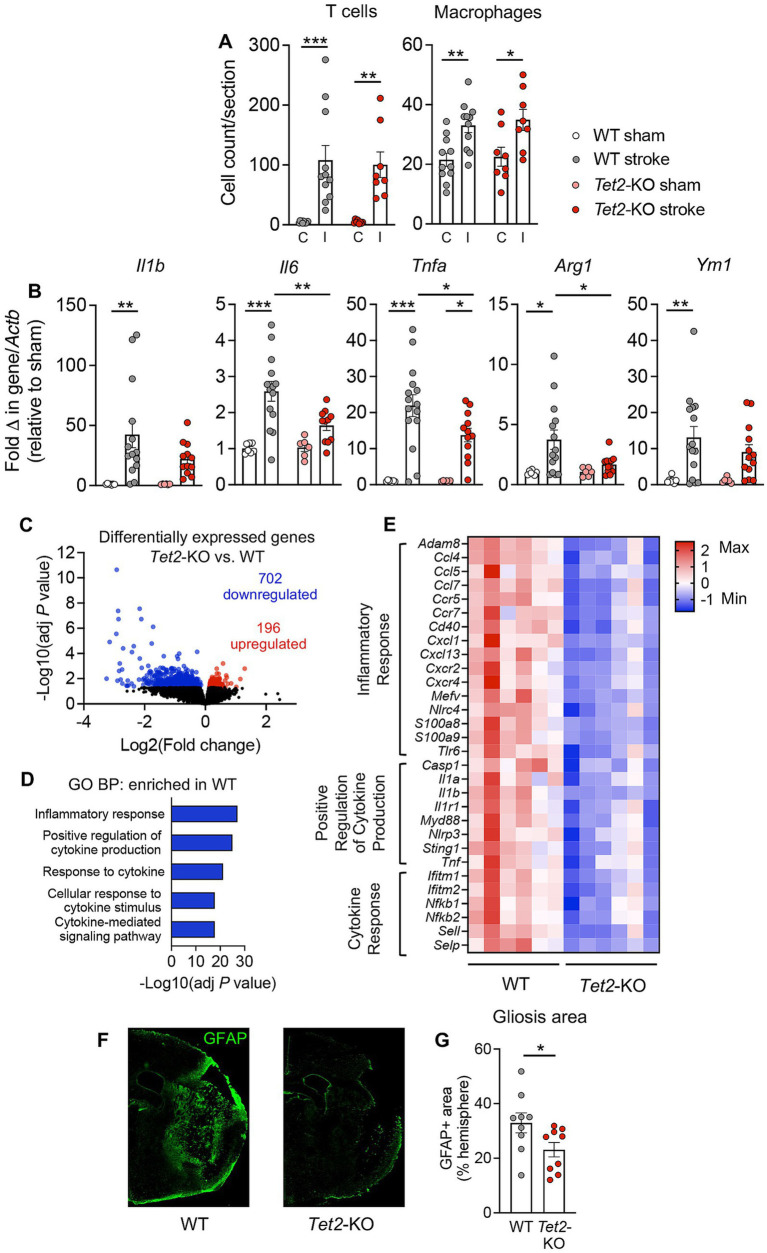
*Tet2*-mediated clonal hematopoiesis is associated with lower levels of brain inflammation and a reduced astrogliosis at 14 d post-stroke. **(A)** Immunohistochemistry was used to determine numbers of CD3+ T cells and F4/80+ macrophages in the ischemic (I) and contralateral (C) brain hemispheres of mice transplanted with wildtype (WT) or *Tet2*-knockout (*Tet2*-KO) bone marrow cells at 14 d post-stroke (WT: *n* = 11 and *Tet2*-KO: *n* = 8). **(B)** qRT-PCR was used to quantify transcriptional expression of inflammatory markers *Il1b*, *Il6*, *Tnfa*, *Arg1* and *Ym1* in the brains of mice transplanted with WT or *Tet2*-KO bone marrow cells at 14 d post-stroke or sham surgery (WT sham: *n* = 7, WT stroke: *n* = 14–15, *Tet2*-KO sham: *n* = 6 and *Tet2*-KO stroke: *n* = 11–13). Bulk RNA-sequencing was used to provide an unbiased analysis of gene expression changes occurring at 14 d post-stroke in mice transplanted with WT and *Tet2-*KO cells. **(C)** Volcano plot showing the number of differentially expressed genes in the brains of mice transplanted with *Tet2*-KO bone marrow cells vs. WT bone marrow cells at 14 d post-stroke (*n* = 6 per group). Downregulated genes shown in blue and upregulated genes shown in red. **(D)** Enriched gene sets identified via Gene Ontology: Biological Process (GO:BP) analysis in the brains of mice transplanted with WT bone marrow cells at 14 d post-stroke. **(E)** Heatmap of significantly downregulated genes in the brains of mice transplanted with *Tet2*-KO bone marrow cells vs. WT bone marrow cells at 14 d post-stroke (*n* = 6 per group, where each square within a row represents one animal). **(F)** Representative brain images displaying glial fibrillary acidic protein (GFAP) immunofluorescence used to measure the amount of reactive gliosis in mice transplanted with either WT or *Tet2*-KO bone marrow cells. **(G)** Extent of reactive gliosis in the brains of mice transplanted with either WT or *Tet2*-KO bone marrow cells at 14 d post-stroke (WT: *n* = 9 and *Tet2*-KO: *n* = 9). Data are presented as mean ± SEM **(A,B,G)**. Statistical comparisons were made using a two-way ANOVA followed by Sidak post-hoc test **(A,B)**, the Wald test **(C,E)** or Student’s unpaired *t* test **(G)**. *p < 0.05, **p < 0.01, ***p < 0.001.

To understand the potential mechanisms by which *Tet2*-mediated CH leads to a modestly improved functional outcome at 14 d post-stroke, we next examined its effects on inflammation during subacute phase of stroke. Numbers of macrophages (F4/80+ cells) and T cells (CD3+ cells) were similar in the brains of mice transplanted with cells of either genotype (Figure 2A), suggesting no influence of *Tet2*-mediated CH on the infiltration of these immune cell types. However, qRT-PCR revealed that mRNA expression of several key inflammatory markers was lower in the brains at 14 d after stroke of mice that received *Tet2*-KO BM cells (Figure 2B). In particular, it was observed that there was a significantly lower transcript expression of *Tnf*, *Il6* and *Arg1* compared to animals transplanted with WT BM cells (Figure 2B). It was also noted that expression of *Il1b* and *Ym1* were significantly elevated in the brains of mice transplanted with WT cells; however changes in these transcripts did not achieve statistical significance in the brains of mice transplanted with *Tet2*-KO cells (Figure 2B). At 24 h post-stroke we found that numbers of neutrophils and macrophages and the levels of these cytokines were similar in mice transplanted with WT and *Tet2*-KO BM, indicating that the levels of inflammation are initially similar between the two groups acutely after stroke (Supplementary Figures 3A,B).

We also performed bulk RNA-sequencing on whole brains to provide an unbiased view of the changes occurring in gene expression at 14 d post-stroke. Principal component analysis revealed that the response to stroke during subacute phase was different between mice transplanted with *Tet2*-KO and WT BM cells (Supplementary Figure 4). Using a statistical cut-off of adjusted *p* < 0.05, we identified 898 genes that were differentially expressed between the ischemic brains of mice transplanted with *Tet2*-KO BM cells and those transplanted with WT cells (Figure 2C). Among these genes, 702 genes were downregulated, and 196 genes were upregulated (Figure 2C). As the majority of differentially expressed genes were downregulated, we primarily focused on gene sets that were enriched in the brains of stroke animals transplanted with WT BM cells. Consistent with qRT-PCR data, the top five significantly enriched gene sets were those closely associated with inflammation and cytokine pathways (Figure 2D). A heatmap representation of select genes, in the most overrepresented pathways, that were significantly downregulated is shown in Figure 2E. These downregulated genes included various chemokines (*Ccl4*, *Ccl5*, *Ccl7*, *Cxcl1*, *Cxcl13*), chemokine receptors (*Ccr5*, *Ccr7*, *Cxcr2*, *Cxcr4*), cytokines (*Il1a*, *Il1b*, *Tnf*) and inflammatory transcription factors (*Nfkb1*, *Nfkb2*). Upregulated gene pathways in mice transplanted with *Tet2*-KO cells, alongside all differentially expressed genes are shown in Supplementary Tables 4–7. In accordance with the fewer neurological deficits in mice transplanted with *Tet2*-KO cells there was an enrichment of gene sets involved in neural synaptic function, such “Chemical Synaptic Transmission,” “Modulation of Chemical Synaptic Transmission,” “Anterograde Trans-Synaptic Signaling,” “Nervous System Development” and “Axonogenesis” (Supplementary Table 5). We also noted that the extent of reactive gliosis, as measured by the area of GFAP+ immunofluorescent staining surrounding the lesion, was significantly smaller in mice transplanted with *Tet2*-KO BM cells (Figures 2F,G).

### Stroke does not promote expansion of *Tet2*-mutant leukocytes

It has been suggested that cardiovascular disease and associated inflammation may drive clonal expansion of *Tet2*-mutant hematopoietic cells (Heyde et al., 2021; Cochran et al., 2023; McClatchy et al., 2023). Thus, we tested whether stroke itself could promote expansion of *Tet2*-mutant leukocytes over the 14 d study period. Peripheral blood was collected pre- and 14 d post-stroke or sham surgery in mice transplanted with either *Tet2*-KO or WT BM cells and analyzed for CD45.2 donor cell chimerism. As expected, *Tet2*-KO donor cells expanded in all leukocyte compartments compared to WT cells, however stroke did not accelerate expansion of *Tet2* mutant cells in this experimental setting (Supplementary Figure 5). Specifically, there was no difference in the percentage of CD45.2 *Tet2*-KO donor cell chimerism between animals subjected to stroke- or sham-surgery across all leukocyte subsets (Supplementary Figure 5).

## Discussion

CH is increasingly being acknowledged as a risk factor for cardiovascular disease, where leukocytes carrying driver mutations can causally contribute to disease pathogenesis (Jaiswal et al., 2014; Fuster et al., 2017; Sano et al., 2018a, b, 2019; Yura et al., 2021; Fuster et al., 2020; Liu et al., 2023; Yu et al., 2023). While ischemic stroke is predominantly driven by a culmination of cardiovascular risk factors; the outcomes are largely neurological, affecting brain tissue and consequently neurological function. Studies suggest that CH is associated with increased ischemic stroke risk (Jaiswal et al., 2014; Bhattacharya et al., 2021), although less is known about the relationship between CH and stroke outcome. Therefore, using *Tet2* as a test driver gene mutation, the current study assessed the impact of CH on acute and subacute outcomes after experimental ischemic stroke.

Here we found that *Tet2*-mediated CH had no effect on acute outcome but led to a modest improvement in neurological function at 14 d post-stroke. Mice transplanted with *Tet2*-KO BM showed a significant improvement in their neurological deficit score from 3 to 14 d post-stroke, and their scores were significantly lower than mice transplanted with WT BM cells. In association with fewer neurological deficits, we found that mice transplanted with *Tet2*-KO cells had a lower level of inflammation in the brain at 14 d post-stroke. Both quantitative RT-PCR and bulk RNA-sequencing revealed that gene expression of inflammatory markers was lower in the brains of mice transplanted with *Tet2*-KO BM cells compared to mice transplanted with WT cells. These included a vast array of genes, such as cytokines, chemokines, chemokine receptors and inflammatory transcription factors. It is noteworthy that at 24 h post-stroke we saw no detectable differences in the expression of inflammatory mediators between the two groups, suggesting that *Tet2*-mediated CH may promote inflammation resolution between the acute and subacute phases of stroke.

In association with lower levels of brain inflammation at the subacute timepoint, it was also observed that the extent of reactive astrogliosis surrounding the lesion was smaller in mice transplanted with *Tet2*-KO BM cells. This reduction astrogliosis occurred in the absence of a change in lesion volume, which has been noted by others previously (Ito et al., 2019; Laterza et al., 2018). Astrocytes are known to detect and respond to pro-inflammatory cytokines released by both resident and infiltrating immune cells (Linnerbauer et al., 2020). Further, inflammatory molecules have been reported to promote the formation of reactive and neurotoxic astrocytes (Giulian et al., 1988; Liddelow et al., 2017; Qian et al., 2007). Thus, it is conceivable that the reduction in astrogliosis observed in mice transplanted with *Tet2*-KO BM cells is a response to the lower level of inflammation in the brain at this timepoint.

It is well established that unresolved chronic inflammation is associated with poorer outcomes after a stroke. Neuronal tissue in the peri-infarct area is particularly vulnerable to the effects of chronic inflammation, where inflammatory processes may impair neural function and synaptic plasticity necessary for functional recovery (Shi et al., 2019). In particular, high levels of certain cytokines, such as IL-1β, TNF, and IL-6, released during injury can impair processes essential for synaptic plasticity (Lynch, 2015; Di Filippo et al., 2008; Di Filippo et al., 2013; Golia et al., 2019). In the current study, it was observed that animals transplanted with *Tet2*-KO cells had lower transcript levels of these cytokines and reduced astrogliosis in the brain at 14 d post-stroke. Concurrently, there was an enrichment of gene sets involved in synaptic function and repair, such as “Chemical Synaptic Transmission,” “Modulation of Chemical Synaptic Transmission,” “Anterograde Trans-Synaptic Signaling,” “Nervous System Development” and “Axonogenesis.” From these data it could be hypothesized that *Tet2*-mediated CH accelerates inflammation resolution after a stroke, facilitating the restoration of synaptic function and improving neurological outcomes. Intriguingly, it was recently reported that CH-positive patients exhibit an increased risk of first myocardial infarction, but not an increased risk of recurrent myocardial infarction (Marston et al., 2024). Perhaps it could be speculated that the increased resolving capacity of *TET2* mutant leukocytes could be protective against a secondary infarction. Nevertheless, future studies are required to better understand this relationship.

Our findings in this mouse model of stroke contrast with the traditional paradigm of CH, where a multitude of studies, including those from our group, suggest that hematopoietic driver mutations promote cardiovascular disease by bolstering inflammatory processes in leukocytes (Jaiswal et al., 2017; Fuster et al., 2017; Sano et al., 2018a; Sano et al., 2018b; Yura et al., 2021; Fuster et al., 2020; Sano et al., 2019; Polizio et al., 2024). The mechanisms underlying this discrepancy remain elusive, although perhaps the injurious mechanisms by which *Tet2*-mediated CH promotes disease in the periphery are less deleterious to the brain. In this regard, hematopoietic *Tet2*-deficiency has been shown to generate a population of macrophages with an augmented inflammatory profile, with elevations reported in numerous cytokines, chemokines, and immune receptors (Jaiswal et al., 2017; Agrawal et al., 2022; Fuster et al., 2017; Sano et al., 2018a, b; Cull et al., 2017). In experimental models of cardiovascular disease, studies indicate that *Tet2*-deficient macrophages promote disease via enhanced production of IL-1β through the NLRP3 inflammasome (Fuster et al., 2017; Sano et al., 2018b; Fuster et al., 2020; Polizio et al., 2024). However, while the NLRP3 inflammasome is elevated in the post-stroke brain, it has been reported that it has minimal contribution to brain injury and neurological function after experimental ischemic stroke (Lemarchand et al., 2019; Denes et al., 2015; Chung et al., 2018). The negligible contribution of the NLRP3 inflammasome to stroke-induced brain injury, may in part, explain why *Tet2*-mediated CH did not worsen ischemic stroke outcomes in the current study.

The beneficial actions of *Tet2*-mediated CH on stroke outcome may be more reflective of the protective role that bloodborne pro-inflammatory macrophages and their monocyte precursors play in ischemic stroke (Chu et al., 2015; Pedragosa et al., 2020; Miró-Mur et al., 2016; Fang et al., 2018; Wattananit et al., 2016; Garcia-Bonilla et al., 2018). Indeed, CCR2+ monocytes have been reported to reduce brain injury, lower inflammation and promote repair in the brain after cerebral ischemia (Chu et al., 2015; Pedragosa et al., 2020; Miró-Mur et al., 2016; Fang et al., 2018; Wattananit et al., 2016). Moreover, post-stroke administration of LPS-primed bone marrow derived monocytes has been shown to reduce brain injury and inflammation (Garcia-Bonilla et al., 2018). These protective actions appear to depend on the production of anti-inflammatory mediators, such as arginase-1 and IL-10 (Chu et al., 2015; Miró-Mur et al., 2016; Garcia-Bonilla et al., 2018), which are produced in high amounts by these cells. Interestingly, it has been documented that while stimulated *Tet2*-deficient macrophages express higher levels of proinflammatory mediators compared to wildtype macrophages, they also express higher levels of inflammation resolving mediators such as *Arg1*, *Il10*, *Socs1*, *Socs3* and *Traf1* (Cull et al., 2017). It is tempting to speculate that upon infiltrating the brain, inflammatory monocytes/macrophages generated through hematopoietic *Tet2*-loss-of-function, produce mediators which more strongly influence inflammation resolution pathways in the ischemic brain than in other tissues or disease settings. Although future work is required to test this hypothesis. It also is important to note, that in the current study we did not observe higher transcriptional levels of pro-resolving molecules in the brains of animals transplanted with *Tet2*-KO cells at 14 d post-stroke. A caveat of our study was that for the gene transcriptional analyses we used whole brain hemispheres, which likely limited our ability to detect mediators produced locally by small numbers of infiltrating mutant cells. Instead, we predict that the inflammatory transcriptional signal that we detected was from parenchymal brain cells (i.e., neurons, astrocytes and microglia) and was a reflection of their response to the mediators released by infiltrating immune cells. Future experiments enabling single-cell transcript analysis could offer deeper insights into the molecular mechanisms by which *Tet2*-mutant cells interact with parenchymal cells to reduce inflammation.

Notwithstanding, there is some, albeit limited, evidence to suggest that CH may have protective actions in other neurological diseases. Firstly, it has been documented that CH is associated with a lower risk of Alzheimer’s disease in individuals without overt cardiovascular disease (Bouzid et al., 2023). Consistently, a published abstract by Wathan et al. reported that bone marrow mutations in *Tet2* improved cognitive function and reduced plaque burden in a mouse model of Alzheimer’s disease (Wathan et al., 2022). Additionally, a preprint has reported that the prevalence of CH is lower in individuals with Parkinson’s disease than in healthy controls (Sun et al., 2023). It has also been observed that individuals with myelodysplastic syndromes and chronic myelomonocytic leukemia have a lower incidence of neurological diseases, including Alzheimer’s and Parkinson’s disease (Weeks et al., 2022). While the mechanisms by which CH leads to a lower incidence of neurological disorders remain to be fully elucidated, one group has posited that in the setting of Alzheimer’s disease, myeloid cells with driver mutations may be more likely to expand in the brain, integrate the microglial niche and aid with the phagocytic clearance of waste and debris (Bouzid et al., 2023). Consistently, following ischemic stroke, phagocytic clearance of dead cells and debris has been shown to promote inflammation resolution and improve neurological outcomes (Nakahashi-Oda et al., 2021; Zhang et al., 2019). Perhaps a similar mechanism may account for the lower level of inflammation observed at the 14 d post-stroke time point in mice transplanted with *Tet2*-KO BM cells, although this warrants further investigation.

While this study represents an important first step in better understanding the relationship between CH and stroke outcome, it is imperative to consider the broader clinical implications of this work. Here we intentionally assessed the impact of *Tet2*-mediated CH on stroke outcome using a mouse model that is void of the confounding effects of underlying cardiovascular disease. It should be noted that many ischemic stroke patients also have underlying cardiovascular risk factors and comorbid conditions, particularly atherosclerosis, hypertension and diabetes (Tsao et al., 2023), and evidence suggests that hematopoietic driver mutations causally contribute to these conditions (Jaiswal et al., 2017; Zekavat et al., 2023; Fuster et al., 2017, 2020; Liu et al., 2023). Thus, it is plausible that the presence of such comorbidities in combination with CH would yield different clinical outcomes in human stroke patients, particularly as heightened risk factors and comorbidities can be associated with unfavorable outcomes (Sennfält et al., 2020; Gallacher et al., 2019; Li et al., 2021). On this point, a small scale clinical study has suggested that ischemic stroke patients with CH have a worse NIH stroke score and 90 d functional disability than patients without CH (Lee et al., 2023). Furthermore, as many ischemic stroke patients will experience a recurrent stroke in their lifetime (Tsao et al., 2023), it would seem prudent to avoid strategies that may exacerbate these risk factors. Therefore, building upon the current findings, future experimental studies could investigate the impact of CH on stroke outcome using models which also possess comorbid conditions, such as diabetes, atherosclerosis and hypertension, individually or in combination, to better understand these complex relationships.

In addition to evaluating the impact of *Tet2*-mediated CH on stroke outcome, we also examined the effect of stroke on expansion of *Tet2*-mutant peripheral blood cells (i.e., reverse causality). Over this relatively short experimental time course, stroke did not promote expansion of *Tet2*-KO donor cells in any leukocyte subset within the blood. Some studies have suggested that cardiovascular disease and inflammation may promote expansion of *Tet2*-mutant leukocytes in the peripheral blood (Heyde et al., 2021; Cochran et al., 2023; McClatchy et al., 2023). However, peripheral inflammation generated as a consequence of stroke, was clearly insufficient to promote expansion in the current setting. It has been discovered that the skull BM is an important reservoir of myeloid cells, which infiltrate the brain following injury and disease (Herisson et al., 2018; Cai et al., 2019; Cugurra et al., 2021). Studies suggest that this niche is maintained with minimal support from the systemic immune system and likewise does not contribute to peripheral immune responses, raising the notion that it is designed to respond to brain-specific injuries (Cugurra et al., 2021). It has also been demonstrated that following CNS injury, the cerebral spinal fluid (CSF), can access the skull BM through channels in the dura where inflammatory mediators signal to resident cells, promoting myelopoiesis and egress (Mazzitelli et al., 2022). It would be interesting to know if skull BM resident leukocytes with driver gene mutations respond differently to stroke or if inflammatory mediators within the CSF promote selective expansion of mutant cells within the skull BM after stroke. As our CH model was created by shielding the head, thereby limiting skull BM engraftment, our study was unable to address these questions.

We acknowledge some limitations of the mouse stroke model we used for these studies. In particular, the sensitivity of the Pep Boy mouse strain to cerebral ischemia may have led to some phenotypic differences in the MCAO stroke model that are not typically observed by other groups. Firstly, our pilot work found that using filaments with longer coating lengths (i.e., 5–6 mm) led to a substantial level of death within the first 24 h post-stroke. Due to the relatively long experimental window required for the mutant cells to expand to reach an adequate level of chimerism (8–10 w), and in attempt to reduce animal usage, we wanted to avoid such a high level of mortality for these studies. Thus, we shortened the coating length of the filaments (1–2 mm) and observed a lower mortality rate at 24 h post-stroke. We also found that 30 min of ischemia was sufficient to produce a sizeable infarct spanning both the subcortical and cortical regions, whereas others have noted that this occlusion time is insufficient in other mouse strains (Chiang et al., 2011; Liesz et al., 2009). Further, we observed that a portion of mice within this strain presented with longer lasting neurological deficits, such as forelimb weakness and circling at the 14 d timepoint, whereas other groups suggest these deficits are acutely resolved (Ruan and Yao, 2020; Li et al., 2004). Despite these strain-based anomalies, we are confident that our analyses of the effect of *Tet2*-mediated CH on murine ischemic stroke outcomes are thorough as the improvement in neurological deficit score is consistent with the reduction in astrogliosis and diminished inflammation revealed by nonbiased transcriptomic analyses at the 14 d timepoint.

In conclusion, findings from this study suggest that in experimental ischemic stroke, without confounding cardiovascular risk factors, *Tet2*-mediated CH leads to a modest improvement in neurological outcome during the subacute phase. This improved outcome is associated with lower levels of inflammation, perhaps indicative that *Tet2*-mutant leukocytes promote inflammation resolution after stroke, allowing for neurological recovery. It is still unclear if the beneficial effects of *Tet2*-mediated CH on stroke outcomes would differ in individuals with cardiovascular risk factors, particularly atherosclerosis and hypertension, which are aggravated by hematopoietic *Tet2* mutations (Fuster et al., 2017; Polizio et al., 2024). Similarly, it is unknown as to whether other driver gene mutations may have similar effects in ischemic stroke. Nevertheless, these findings are proof-of-concept that CH driver mutations can have diverse effects on disease outcomes. While in many diseases they may lead to pathology, in others they could be benign or even offer beneficial effects.

## Data Availability

Bulk raw sequencing data and processed data files used to generate and perform analyses of post-stroke brains are available in the NCBI GEO repository under accession no. GSE270710. All other data presented in the manuscript will be made available by authors upon reasonable request.
