# Supplementary material for: Tet2-mediated clonal hematopoiesis modestly improves neurological deficits and is associated with inflammation resolution in the subacute phase of experimental stroke
Source: Front Cell Neurosci. 2024 Dec 17;18:1487867. doi: 10.3389/fncel.2024.1487867 (PMC11685025; doi:10.3389/fncel.2024.1487867)
Supplement: Supplementary file 2 [file Data_Sheet_2.pdf]

Data supplement

Immunofluorescent staining

Six serial coronal sections (10 µm thick) per animal were collected onto poly-l-lysine coated slides at six regions: Bregma + 0.06, − 0.78, − 1.2, − 1.62, − 2.04, − 2.46 mm. Sections were stored at -80 °C until required. Frozen brain sections (10 µm) were fixed with 4% paraformaldehyde for 15 min and washed in 0.01 M PBS (3 × 10 min). Sections were then blocked with 10% goat serum (Abcam; diluted in PBS) for 1 h. Sections were subsequently incubated with primary antibodies (listed **Supplementary Table 3**) diluted in antibody diluent (0.2% Triton X-100 in 0.01 M PBS), overnight at room temperature. The following day, sections were washed (0.01 M PBS; 3 × 10 min) and incubated for 2 h with appropriate secondary antibodies (listed **Supplementary Table 3**). Finally, sections were washed (0.01M PBS; 3 x 10 min), mounted with Vectashield Plus Antifade Mounting Medium containing 4,6-diamidino-2-phenylindole (DAPI) (Vector Laboratories, CA, USA), and a coverslip was applied. Tissue-mounted slides were viewed, analyzed and/or photographed with either a Keyence (BZ-X710; Keyence Co, IL, USA), Olympus (BX53; Olympus Corp, Japan) or Leica (Thunder Live Imager; Germany) fluorescence microscope. For immune cell counts, numbers of immunoreactive cells per ischemic or contralateral hemisphere were counted manually under the microscope (20x magnification) and then averaged across the six brain regions. For gliosis measurements, whole brain sections (Bregma +0.06 and -0.78) were tile scanned at 4x magnification and images were reconstructed using BZ-X710 analysis software. To determine the extent of reactive gliosis, the GFAP+ area immediately adjacent to the lesion was measured using ImageJ software. Sections were, usually and wherever possible, analyzed in a blinded manner.

Bulk RNA-sequencing

Sequencing

RNA samples were firstly quantified using Qubit 2.0 Fluorometer (Life Technologies, CA, USA) and RNA integrity was measured using the RNA Screen Tape on Agilent 2200 TapeStation (Agilent Technologies, CA, USA). rRNA depletion was performed using QIAseq® FastSelect™−rRNA HMR kit (Qiagen, MD, USA) according to the manufacturer's protocol. RNA sequencing libraries were constructed with the NEBNext Ultra II RNA Library Preparation Kit for Illumina by following the manufacturer's recommendations. Briefly, enriched RNAs were fragmented for 15 min at 94 °C. First strand and second strand cDNA were subsequently synthesized. cDNA fragments were end repaired and adenylated at 3'ends,

and universal adapters were ligated to cDNA fragments, followed by index addition and library enrichment with limited cycle PCR. Sequencing libraries were validated using the Agilent Tapestation 4200 (Agilent Technologies, CA, USA), and quantified using Qubit 2.0 Fluorometer (ThermoFisher Scientific, USA) as well as by quantitative PCR (KAPA Biosystems, Wilmington, MA, USA). The sequencing libraries were multiplexed and clustered on 1 lane of a flowcell. After clustering, the flowcell was loaded on the Illumina HiSeq 4000 instrument according to manufacturer's instructions. The samples were sequenced using a 2x150 Pair-End (PE) configuration.

Data analysis

After demultiplexing, sequence data was checked for overall quality and yield. Then, sequence reads were trimmed to remove possible adapter sequences and nucleotides with poor quality using Trimmomatic v.0.36. The trimmed reads were mapped to the reference genomes using the STAR aligner v.2.5.2b. The STAR aligner is a splice aware aligner that detects splice junctions and incorporates them to help align the entire read sequences. BAM files were generated as a result of this step. Unique gene hit counts were calculated by using featureCounts from the Subread package v.1.5.2. Only unique reads within exon regions were counted. Using DESeq2, a comparison of gene expression between the groups of samples was performed. The Wald test was used to generate *P* values and Log2 fold changes. Genes with adjusted *P* values <0.05 were determined as differentially expressed genes for each comparison. Gene ontology analysis was performed on the statistically significant set of genes by implementing the EnrichR package in R[1]. The GO list was used to cluster the set of genes based on their biological process and determine statistical significance. A PCA analysis was performed using the "plotPCA" function within the DESeq2 R package. The plot shows the samples in a 2D plane spanned by their first two principal components. The top 500 genes, selected by highest row variance, were used to generate the plot.

Supplementary Table 1

Antibody	Fluorochrome	Clone	Source	Identifier
Anti-CD45.1	PE-Cy7	A20	eBioscience, ThermoFisher	Cat No. 25-0453-82; RRID: AB_469629
Anti-CD45.2	Brilliant-Violet 785	104	BioLegend	Cat No. 109839; RRID: AB_2562604
Anti-CD115	PE	AFS98	eBioscience, ThermoFisher	Cat No.12-1152-82; RRID: AB_465808
Anti-CD3e	PE-eFluor610	145-2C11	eBioscience, ThermoFisher	Cat No. 61-0031-82; RRID: AB_2574514
Anti-CD45R/B220	APC-Cy7	RA3-6B2	BD Biosciences	Cat No. 552094; RRID: AB_394335
Anti-Ly6C	APC	AL-21	BD Biosciences	Cat No. 560595; RRID: AB_1727554
Anti-Ly6G	PerCP-Cy5.5	1A8	BD Biosciences	Cat No. 560602; RRID: AB_1727563

Supplementary Table 2

**Custom Primers**			
Gene Name	Species	Forward 5’ to 3’	Reverse 5’ to 3’
Il1b	*Mus Musculus*	TGACAGTGATGAGAATGACCTGTTC	TTGGAAGCAGCCCTTCATCT
Il6	*Mus Musculus*	GCTACCAAACTGGATATAATCAGGA	CCAGGTAGCTATGGTACTCCAGAA
Tnfa	*Mus Musculus*	CGGAGTCCGGGCAGG	GCTGGGTAGAGAATGGATGAA
Arg1	*Mus Musculus*	CATCAACACTCCCCTGACAAC	GTACACGATGTCTTTGGCAGA
Chi3l3 (Ym1)	*Mus Musculus*	AGGCTGCTACTCACTTCCACA	TTTTCTCCAGTGTAGCCATCC
Actin	*Mus Musculus*	GGCTGTATTCCCCTCCATCG	CCAGTTGGTAACAATGCCATGT

Supplementary Table 3

Primary Antibodies	Source	Identifier
Rabbit anti-myeloperoxidase (1:100)	Abcam	ab9535
Rabbit anti-CD3e (1:200)	Abcam	ab16669
Rat anti-F4/80 (1:100)	Bio-Rad	MCA497G
Rabbit anti-GFAP (1:500)	Abcam	ab7260
**Secondary antibodies**	**Source**	**Identifier**
Alexa Fluor 594 goat anti-rabbit (1:500)	Invitrogen, ThermoFisher Scientific	A11037
Alexa Fluor 488 goat anti-rabbit (1:500)	Invitrogen, ThermoFisher Scientific	A11008
Alexa Fluor 594 goat anti-rat (1:500)	Invitrogen, ThermoFisher Scientific	A11007

References

1. Kuleshov, M.V., Jones, M.R., Rouillard, A.D., Fernandez, N.F., Duan, Q., Wang, Z., Koplev, S., Jenkins, S.L., Jagodnik, K.M., Lachmann, A., et al. (2016). Enrichr: a comprehensive gene set enrichment analysis web server 2016 update. Nucleic Acids Res *44*, W90-97. 10.1093/nar/gkw377.
