# Supplementary material for: Tet2-mediated clonal hematopoiesis modestly improves neurological deficits and is associated with inflammation resolution in the subacute phase of experimental stroke
Source: Front Cell Neurosci. 2024 Dec 17;18:1487867. doi: 10.3389/fncel.2024.1487867 (PMC11685025; doi:10.3389/fncel.2024.1487867)
Supplement: Supplementary file 4 [file Image_1.pdf]

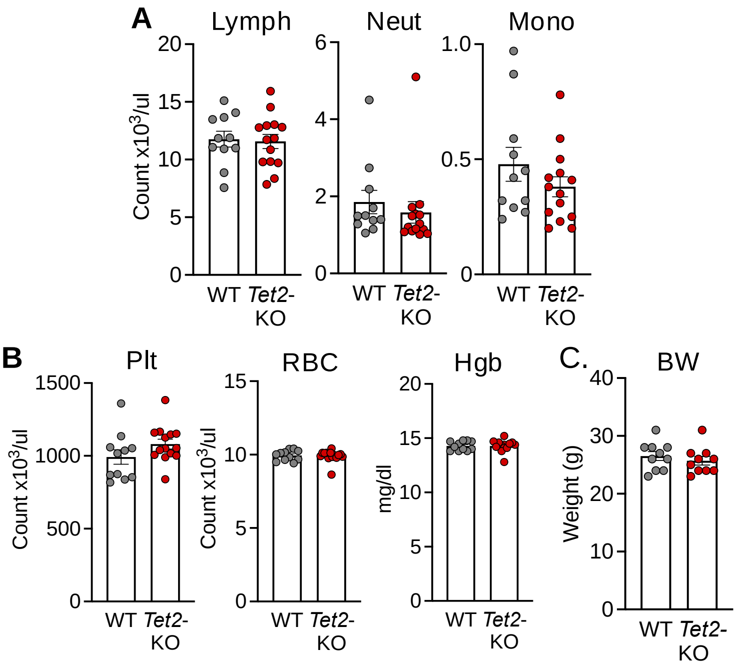

Supplementary Figure 1. *Tet2*-mediated clonal hematopoiesis does not affect hematological parameters or body weight. (A) Peripheral blood counts of lymphocytes (lymph), neutrophils (neut) and monocytes (mono) and (B) levels of platelets (Plt), red blood cells (RBCs) and hemoglobin (Hgb) in mice at 8 weeks post-bone marrow transplant (BMT) with either wildtype (WT) or *Tet2*-knockout (KO) cells (WT: n = 11 and *Tet2*-KO: n = 14). (C) Body weight (BW) of mice at 8 weeks post-BMT with either WT or *Tet2*-KO cells (n = 10 per group). Data are presented as mean ± SEM. Statistical comparisons were made using a Student's unpaired t test.

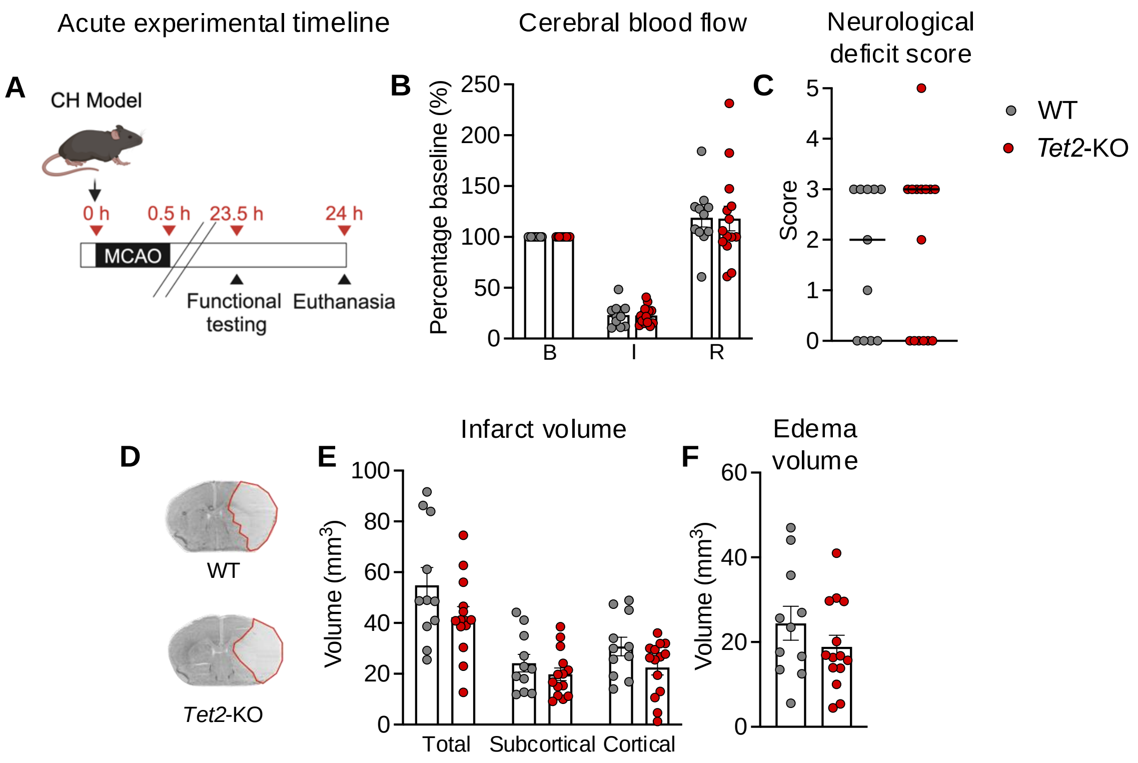

Supplementary Figure 2. *Tet2*-mediated clonal hematopoiesis does not affect outcome at 24 h post-stroke. (A) Experimental timeline for acute stroke experiments. Mice were subjected to middle cerebral artery occlusion (MCAO) to induce stroke and outcomes were assessed 24 h later. (B) Regional cerebral blood flow was recorded prior to (baseline; B), during (ischemia; I) and after (reperfusion; R) stroke in mice transplanted with either wild type (WT) or *Tet2*-knock out (*Tet2*-KO) bone marrow cells (WT: n = 11 and *Tet2*-KO n = 14). (C) Post-stroke neurological deficit scores from mice transplanted with WT or *Tet2*-KO bone marrow cells. (D) Representative coronal brain sections delineating area of infarction (outlined in red) from mice transplanted with either WT or *Tet2*-KO bone marrow cells. (E) Infarct and (F) edema volumes of mice transplanted with either WT or *Tet2*-KO bone marrow cells. WT: n = 11 and *Tet2*-KO: n = 14-15 for all data sets. Data are presented as mean ± SEM except for neurological deficits scores where data are presented as median. Statistical comparisons were made using multiple t tests (B), Mann-Whitney test (C) or Student's unpaired t test (E and F).

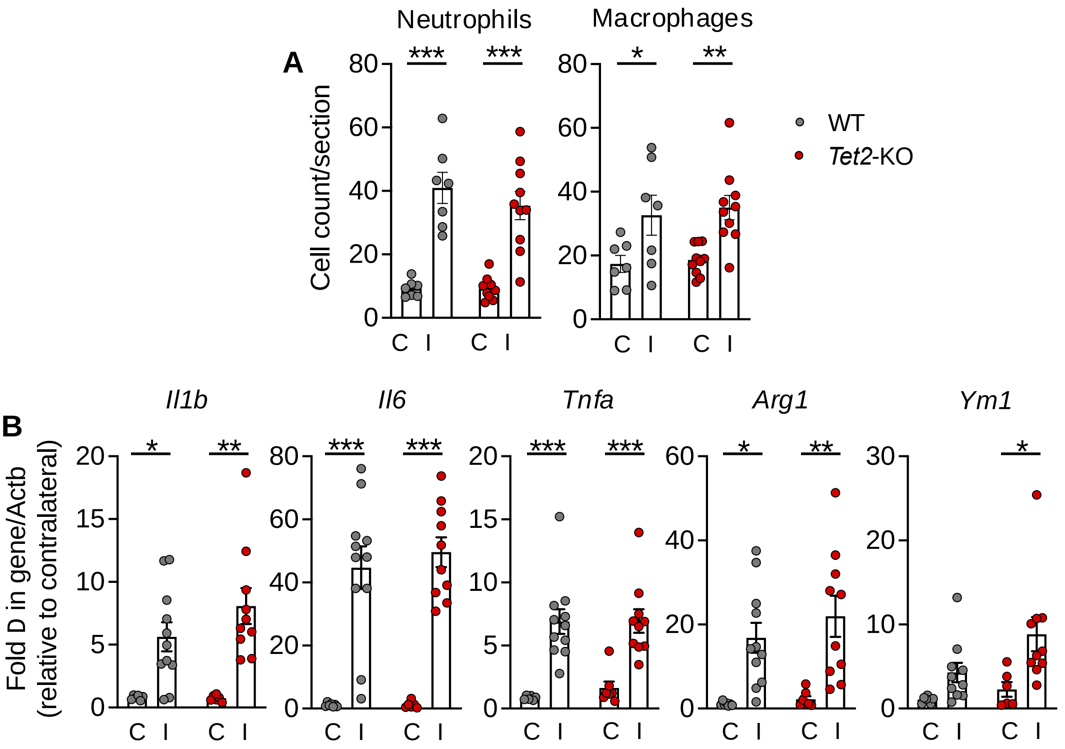

Supplementary Figure 3. *Tet2*-mediated clonal hematopoiesis has no effect on inflammation at 24 h post-stroke. (A) Immunohistochemistry was used to determine numbers of MPO+ neutrophils and F4/80+ macrophages in the ischemic (I) and contralateral (C) hemispheres of mice transplanted with wildtype (WT) or *Tet2*-knockout (*Tet2*-KO) bone marrow cells at 24 h post-stroke (WT: n = 7 and *Tet2*-KO: n = 10). (B) qRT-PCR was used to quantify transcriptional expression of inflammatory markers *Il1b*, *Il6*, *Tnfa*, *Arg1* and *Ym1* in the ischemic (I) and contralateral (C) hemispheres of mice transplanted with WT or *Tet2*-KO bone marrow cells at 24 h post-stroke (WT contralateral: n = 6-7, WT ischemic: n = 10-11, *Tet2*-KO contralateral: n = 6-7 and *Tet2*-KO ischemic: n = 10). Data are presented as mean ± SEM. Statistical comparisons were made using two-way ANOVA followed by Sidak's multiple comparisons test. *P<0.05, **P<0.01, ***P<0.001.

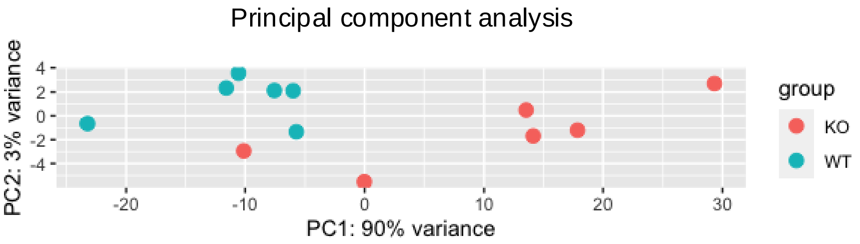

Supplementary Figure 4. Mice transplanted with *Tet2*-knockout bone marrow exhibit a different response to the subacute phase of stroke. Principal component (PC) analysis of bulk RNA-sequencing data obtained from the brains of mice transplanted with wildtype (WT) or *Tet2*-knockout (KO) bone marrow cells at 14 d post-stroke (n = 6 per group).

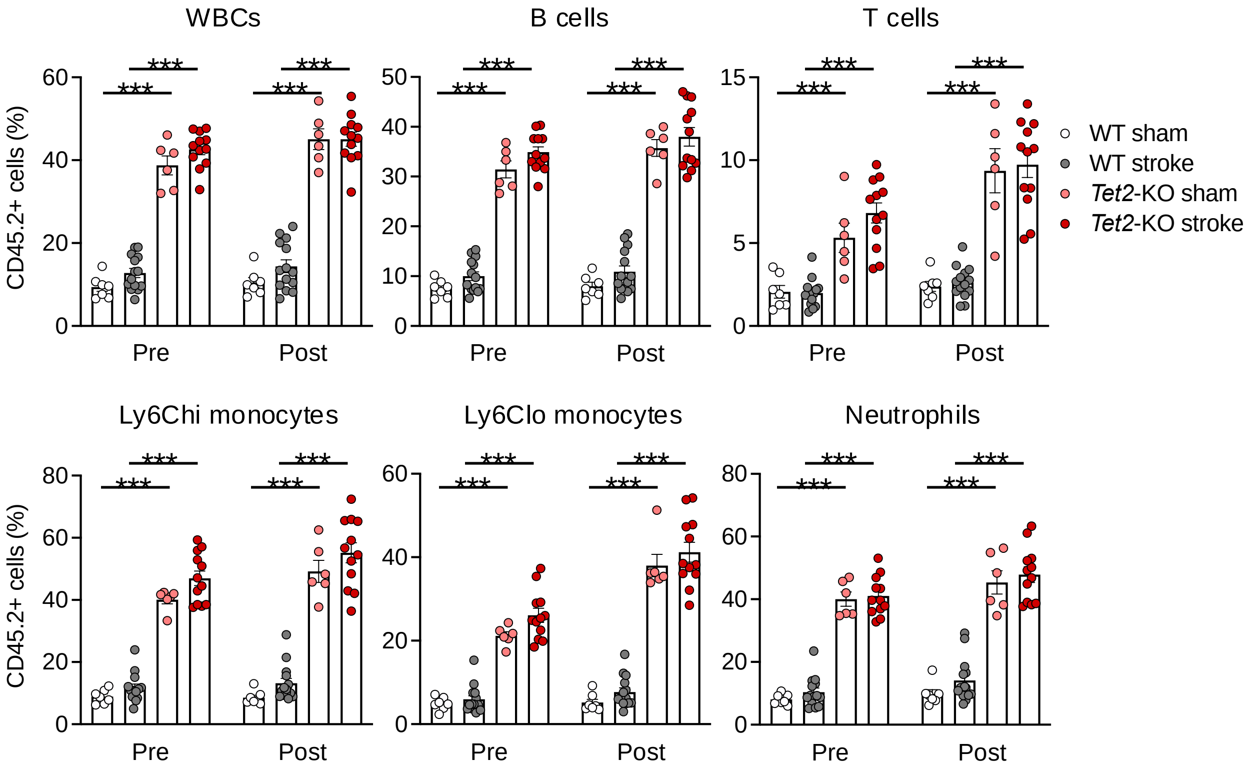

Supplementary Figure 5. *Tet2*-knock out donor cell chimerism does not change in response to stroke. From left-right, top-bottom: Flow cytometric assessment of donor cell chimerism inr white blood cells (WBCs), B cells, T cells, Ly6Chi monocytes, Ly6Clo monocytes and neutrophils from mice transplanted with wildtype (WT) or *Tet2*-knockout (*Tet2*-KO) bone marrow cells pre- and 14 d post-sham or stroke surgery (WT sham: n = 7, WT stroke: n = 14, *Tet2*-KO sham: n = 6 and *Tet2*-KO stroke n = 12). Data are presented as mean ± SEM. Statistical comparisons were made using a two-way ANOVA followed by Sidak's multiple comparison test. ***P<0.001.
